# Selective Autophagy Receptor NBR1 Retards Nucleus Pulposus Cell Senescence by Directing the Clearance of SRBD1

**DOI:** 10.7150/ijbs.90186

**Published:** 2024-01-01

**Authors:** Honghai Song, Yutao Zhu, Chuan Hu, Qianyu Liu, Yang Jin, Pan Tang, Jiechao Xia, Dingqi Xie, Sicheng Jiang, Geliang Yao, Zhili Liu, Zhijun Hu

**Affiliations:** 1 Department of Orthopaedic Surgery, Sir Run Run Shaw Hospital, Medical College of Zhejiang University, Hangzhou 310016, China.; 2 Key Laboratory of Musculoskeletal System Degeneration and Regeneration Translational Research of Zhejiang Province, Hangzhou 310016, China.; 3 Ministry of Education Key Laboratory of Biosystems Homeostasis & Protection, College of Life Sciences, Zhejiang University, Hangzhou 310058, China.; 4 Department of Orthopedic Surgery, The First Affiliated Hospital of Nanchang University, Nanchang 330006, China.; 5 Institute of Spine and Spinal Cord, Nanchang University, Nanchang 330006, China.; 6 Department of Orthopaedics, Affiliated Huzhou Hospital, Zhejiang University School of Medicine, Huzhou 313000, China.

**Keywords:** intervertebral disc degeneration, selective autophagy, NBR1, cellular senescence, SRBD1

## Abstract

Intervertebral disc degeneration (IDD) is a prevalent degenerative disorder that closely linked to aging. Numerous studies have indicated the crucial involvement of autophagy in the development of IDD. However, the non-selective nature of autophagy substrates poses great limitations on the application of autophagy-related medications. This study aims to enhance our comprehension of autophagy in the development of IDD and investigate a novel therapeutic approach from the perspective of selective autophagy receptor NBR1. Proteomics and immunoprecipitation and mass spectrometry analysis, combined with *in vivo* and *in vitro* experimental verification were performed. NBR1 is found to be reduced in IDD, and NBR1 retards cellular senescence and senescence-associated secretory phenotype (SASP) of nucleus pulposus cells (NPCs), primarily through its autophagy-dependent function. Mechanistically, NBR1 knockdown leads to the accumulation of S1 RNA-binding domain-containing protein 1 (SRBD1), which triggers cellular senescence via AKT1/p53 and RB/p16 pathways, and promotes SASP via NF-κβ pathway in NPCs. Our findings reveal the function and mechanism of selective autophagy receptor NBR1 in regulating NPCs senescence and degeneration. Targeting NBR1 to facilitate the clearance of detrimental substances holds the potential to provide novel insights for IDD treatment.

## 1. Introduction

Intervertebral disc degeneration (IDD) is a chronic degenerative disease that is influenced by factors such as age and injury^1^. The nucleus pulposus (NP), a vital constituent of the intervertebral disc, experiences profound structural changes throughout the development of IDD^2^. Nucleus pulposus cells (NPCs) uphold disc height by excreting extracellular matrix (ECM) components, notably the big aggregating proteoglycan aggrecan (ACAN)^3^, ^4^. IDD carries significant implications for both individuals and socioeconomic factors. However, over the past thirty years, the treatment approaches for IDD have exhibited limited advancements.

Cellular senescence emerges as a principal factor driving the occurrence of IDD. The senescence of NPCs is usually induced by age, DNA damage, oncogene and mitochondrial dysfunction^5^, ^6^. The characteristics of senescence encompass irreversible cell cycle arrest, oxidative damage, and the emergence of a senescence-associated secretory phenotype (SASP)^6^, ^7^. Senescent NPCs release chemokines, proinflammatory molecules (such as IL-1β, IL-6), and metalloproteases (MMPs and ADAMTS)^8^, ^9^, which intensify the localized inflammatory microenvironment and ECM degradation metabolism within the nucleus pulposus. These effects significantly accelerate the progression of IDD. Therefore, unraveling the mechanisms underlying NPCs senescence and developing corresponding therapeutic strategies has become a focus of current investigations aimed at improving IDD treatment.

Autophagy, serving as a pivotal process for intracellular quality control and maintenance of cellular homeostasis, has been found to be increased in human NPCs, subsequently influencing the progression of IDD^10^-^12^. Autophagy can be classified into two distinct types based on substrate specificity. Non-selective autophagy, commonly referred to as macroautophagy, is typically triggered in response to specific cellular conditions such as starvation, leading to the indiscriminate degradation of intracellular components. Conversely, selective autophagy represents another form of autophagy that specifically targets cargos with potential harm for degradation^13^. The specificity of this process is contingent upon selective autophagy receptors (SARs). These receptors engage in pattern recognition and bridge the identified substrates to the autophagosome for subsequent degradation^13^, ^14^. Despite compelling evidence indicating the important role of non-selective autophagy in IDD, the contribution of selective autophagy to IDD remains poorly understand.

Neighbor of BRCA1 (NBR1) is one of the known members of the selective autophagy receptor (SAR) family. The structural features of NBR1 encompass the presence of (1) two LC3 interacting regions (LIR), which serve to bind to the autophagosomal membrane component LC3; (2) a ubiquitin-associated (UBA) domain for engaging ubiquitinated substrates^15^, ^16^. Drawing upon its ability to mediate autophagosomal clearance, NBR1 has been reported to exert a significant role in regulating protein aggregate formation^17^, tumor cell metastasis^18^, and immune evasion^19^. In addition, researches have indicated that NBR1 is involved in processes that are independent of autophagy. For instance, Merkley et al. reported that NBR1 participates in delivering IL-12 to late endosomes, thereby countering IFN-γ-driven intestinal inflammation^20^. Despite these findings, the role of NBR1 in age-related chronic degenerative diseases such as IDD remains undisclosed.

In this study, we present, for the first time, the significant role of selective autophagy receptor NBR1 in regulating senescence of NPCs and intervertebral disc degeneration. Our findings indicate that the expression of NBR1 is reduced in degenerated NPCs. NBR1 knockdown drives NPCs senescence through its autophagy-dependent functionality. Furthermore, through proteomic combined with immunoprecipitation and mass spectrometry analysis, we successfully identified RNA-binding protein SRBD1 as a novel substrate of NBR1. In conclusion, our study elucidates a novel and critical role of NBR1 in modulating the processes of IDD through selective autophagy.

## 2. Materials and Methods

### 2.1 Human NP tissues collection

Human nucleus pulposus tissues were obtained from 45 patients with spinal deformities, thoracolumbar vertebral fractures, or intervertebral disc degeneration who underwent disc excision surgery. All intervertebral disc samples were graded according to the Pfirrmann classification system^21^. Patients with Pfirrmann grades Ⅰ and Ⅱ were included as the normal group (n=11). Patient with Pfirrmann grades Ⅲ, Ⅳ, and Ⅴ were included as the IDD group (n=34).

### 2.2 Human NPCs isolation and culture

All surgically excised nucleus pulposus samples were transported and processed within a sterile environment. After washed with PBS, the samples were sectioned into small fragments and subjected to digestion at 37°C for 4-6 hours using 0.2% type II collagenase (9001-12-1, Sigma, USA). The tissue suspension was centrifuged to remove the supernatant, then the collected cells were resuspended in Dulbecco's modified Eagle's medium (DMEM, CR12800-S, Cienry, China) containing 10% FBS (NFBS2500a, Noverse, Australia) and 1% penicillin/streptomycin, and cultured in incubator with 5% CO_2_ at 37°C. Experiments were conducted using NPCs at passages 2 or 3.

### 2.3 Plasmid construction and lentiviral transduction

For RNA interference, relative shRNA plasmids were constructed with pLV-U6-T2A vector (Hanbio, China), the sequences were list as follows: *NBR1* shRNA #1, 5′-GCTTCATAGTTATTTGGCATT-3′, *NBR1* shRNA #2, 5′-GCAGCATTTGTGGATGAGAAT-3′; *SRBD1* shRNA #1, 5′-GCATCAATCTACAGTGTCA-3′, *SRBD1* shRNA #2, 5′-GAATGACGATGACTTTACA-3′; SQSTM1 shRNA #1, 5′-GGAGTCGGATAACTGTTCA-3′, SQSTM1 shRNA #2, 5′-TGAGGAAGATCGCCTTGGA-3′; TAX1BP1 shRNA #1, 5′-CCTCTACTGTAGATGTAAA-3′, TAX1BP1 shRNA #2, 5′-CAGTGATGCTGTCAACGTA-3′. For NBR1 overexpression, the full-length and UBA-encoded region-deleted *NBR1* cDNA were synthesized and inserted into pLV-CMV-Flag vector. The plasmids were transfected with packing plasmids into 293T cells using Lipofectamine 3000 (L3000015, Invitrogen) to generate lentiviruses. The collected viral supernatant was added into NPCs with polybrene (H8761, Solarbio, China) to obtain stable mass transfectants.

### 2.4 Alcian blue and senescence β‐galactosidase (SA‐β‐Gal) staining

For Alcian blue staining, 4×10^6^ infected NPCs were suspended with 20 μL DMEM medium and added into 12-well plate for adhesion in incubator. 2 hours later, 1 mL of DMEM medium was added, and the cells were cultured for an additional 4 days. Finally, the cells were stained with Alcian blue solution (G1560, Solarbio) and photographed. Origin images of Alcian blue were then shifted by ImageJ (Version 1.54F, National Institutes of Health, USA) on same parameters and quantified.

For cellular senescence detection, infected NPCs were stained with SA‐β‐Gal staining kit (C0602, Beyotime, China) according to the manufacturer's instructions. The SA‐β‐Gal positive cells were observed by microscope (Leica, Germany) and counted by ImageJ software.

### 2.5 CCK8 assay

Cell proliferation ability was evaluated by CCK8 assay. 2000 infected NPCs were seeded in the 96-well and cultured for 1, 2, 3, 4 days, respectively. The Cell Counting Kit (CK04, Dojindo, Japan) was added into the medium. After 2 hours incubation, the cell viability was measured by microplatereader at 450 nm.

### 2.6 Flow cytometry

Cell cycle, apoptosis assays were determined by flow cytometry. Briefly, the infected NPCs were digested and stained with PI/Rnase staining buffer (550825, BD, USA) and FITC/Annexin-Ⅴ apoptosis buffer (559763, BD) for cell cycle and apoptosis detection, respectively. The cell phase and apoptosis rate were analyzed by FACSCanto Analyzer (BD).

### 2.7 Intracellular reactive oxygen species (ROS) detection

Briefly, the infected NPCs were stained with DCFH-DA buffer (CA1410, Solarbio) for 30 minutes and imaged by confocal microscope (A1-HD25, Nikon, Japan). For flow cytometry, the NPCs were digested and incubated with DCFH-DA buffer for 30 minutes at 37℃. Then the cellular ROS level was analyzed by FACSCanto Analyzer.

### 2.8 Western blot (WB)

NPCs were lysed with RIPA lysis buffer (FD009, Fudebio, China) containing 1% PMSF and mixture of protease inhibitor cocktails (FD1001, Fudebio). The protein samples were electrophoresed by SDS-PAGE and transferred onto 0.22 μm polyvinylidene difluoride (PVDF) membranes. After blocking with 5% milk (FD6006, Fudebio) for 1 hours, the membranes were incubated with primary antibody at 4℃ overnight. The antibodies information was listed in **Table S2**. After washing three times with TBST, the membranes were incubated with the corresponding secondary antibody for 1 hour. Finally, the bands were visualized using ChemiDoc imaging system (#1708370, Bio-Rad, USA).

### 2.9 RNA extraction and quantitative real-time PCR (qRT-PCR)

Total cellular RNA of NPCs was extracted using TRIzol reagent (T9108, TaKaRa, Japan). PrimeScript RT Reagent (AG11705, AG, China) was used for reverse transcription. The cDNA was amplified with primers and Hieff qPCR SYBR mix (11201ES, Yeason, China) using ABI 7500 sequencing detection system. The primers were listed in **Table S1**. β-actin was used as reference gene.

### 2.10 4D label-free proteomics analysis

The 4D label-free proteomics analysis was supported by Applied Protein Technology (China). Briefly, NPCs infected with shNBR1 or shNC lentivirus were collected for label-free proteome analysis. 20 µg of protein was separated on a 12.5% SDS-PAGE gel and visualized by Coomassie Blue staining. Then the bands were digested into peptides using trypsin, followed by desalting and concentrating by vacuum centrifugation. The reconstituted peptides were then subjected to liquid chromatography-tandem mass spectrometry (LC-MS) analysis. The MS raw data for each sample were combined and searched using the MaxQuant software for identification and quantitation analysis. Proteins with |log_2_FC (shNBR1/shNC) | > 1 and *p*-value < 0.05 were considered as differentially expressed. Gene ontology (GO), Kyoto Encyclopedia of Genes and Genomes (KEGG) pathway enrichment analysis and Gene set enrichment analysis (GSEA) were performed by OmicStudio tool (https://www.omicstudio.cn). Protein-Protein Interaction (PPI) analysis were performed using STRING database (https://www.string-db.org/).

### 2.11 Immunoprecipitation and mass spectrometry (IP-MS)

Proteins were prepared using the method described in the preceding WB section. 10% of the obtained protein was set as the input. The remaining was incubated with antibodies and Protein A/G beads (88803, Invitrogen) at 4°C overnight. The beads were resuspended in 1× loading buffer and boiled to obtain the immunoprecipitants. For mass spectrometry, the proteins were separated by SDS-PAGE and visualized by silver staining (G7210, Solarbio). The gels were then enzymatically disintegrated and analyzed by Applied Protein Technology (China). Protein incubated with IgG antibody (3900S, Cell signaling technology, USA) was defined as control group.

### 2.12 Animal model

The coccygeal IDDs needle stab (CINS) model was established to simulate intervertebral disc degeneration *in vivo*. After the induction of anesthesia using inhaled isoflurane, a needle (27-G) was vertically inserted into the fibrous annulus of the intervertebral disc between Co6-Co7 of the SD rat (aged 3 months) tail, rotated 180°, and left in place for 30 seconds. For the sham group, only a needle puncture through the skin was performed. 4 weeks later, the rats were euthanized, and the tails were collected for subsequent experiments. For the adeno-associated viral vector (AAV) infection, the microliter syringe (87943, Hamilton, Switzerland) with a 33-G needle was used to stab into the fibrous annulus, and a total of 3μL AAV were intro-disc injected. 4 weeks post-surgery, all rats were anesthetized and underwent imaging examinations using a 1.5-T clinical magnet (MR5300, Philips, Netherlands) and high-resolution μCT scanner (Skyscan1275, Bruker, Belgium). The degeneration level of rat intervertebral disc was accessed by Pfirrmann grade system and disc height index (DHI) changes^22^.

### 2.13 Behavioural testing

The pain phenotype of rat intervertebral disc injury was determined by Hargreaves and Von Frey test^23^. For Hargreaves test, rats were individually placed in opaque experimental boxes for half an hour to remove environmental interference. After acclimatization, the puncture site of rat tail was irradiated using a radiant light source. The thermal withdrawal latency of rat was calculated to assess the thermal nociceptive threshold. The experiment was conducted on postoperative days 7 and 14, with three measurements taken for each rat at each time point. The average response time was calculated and used as the final value.

For Von Frey test, rats were individually placed in opaque experimental boxes for half an hour to remove environmental interference. After acclimatization, Von Frey filaments were applied to the tails of mice with pressure. A positive response was recorded when the mouse exhibited behaviors such as licking, flinching, or tail shaking in response to the stimulus. The filaments of different weights were used for measurement five times each, filament weight at which a 50% positive response occurred was recorded as the mechanical pain threshold. This experiment was conducted on postoperative days 7 and 14. Each measurement on the same day was separated by an interval of at least 1 minute.

### 2.14 Histological and immunostaining assays

Human NP tissues were fixed in 4% paraformaldehyde (PFA) for 48 hours, followed by embedding and sectioning into 5μm slices. Rat tail samples (Co6-7, 7-8 discs) were fixed in 4% PFA for 48 hours and decalcified using 10% EDTA for 30 days. The sections were stained by hematoxylin and eosin and safranin fast green (S&O, S8884, Sigma) solutions. Histological scores of rats intervertebral discs were calculated as previously described (3, normal intervertebral disc; 6, moderate IDD; 9, severe IDD)^24^. Immunohistochemistry (IHC) assay was performed using IHC kit (CW2069S, Cwbio, China) according to manufacturer's instructions.

For Immunofluorescence (IF) staining, sections and cells were permeabilized by Triton X-100 (T8787, Sigma) and blocked by 5% bovine serum albumin (BSA), followed by incubation of primary antibodies (Table S2) at 4 ℃ overnight. Then the sections were washed with PBS and incubated with anti-mouse/rabbit Alexa Fluor 488 or 568 secondary antibodies for 30 minutes. Finally, the sections were imaged by digital pathology scanner (KF-FL-005, KFBIO, China) or confocal microscope. The fluorescence intensity was evaluated by ImageJ software.

### 2.15 Statistical analysis

All data are presented as the mean ± SD from at least three independent experiments. Statistical analyses were performed with GraphPad Prism software (Version 9.0, GraphPad, USA), The normality of data distribution was assessed using the Kolmogorov-Smirnov test. Student's t-tests and one-way analysis of variance (ANOVA) followed by Tukey's test were performed for comparisons between two or more groups. Non-parametric data (Pfirrmann grades and S&O staining intensity of human samples) were analyzed using the Mann-Whitney U test. A *p*-value < 0.05 was considered statistically significant.

## 3. Results

### 3.1 NBR1 expression is downregulated in degenerative NPCs

**Figure 1:**
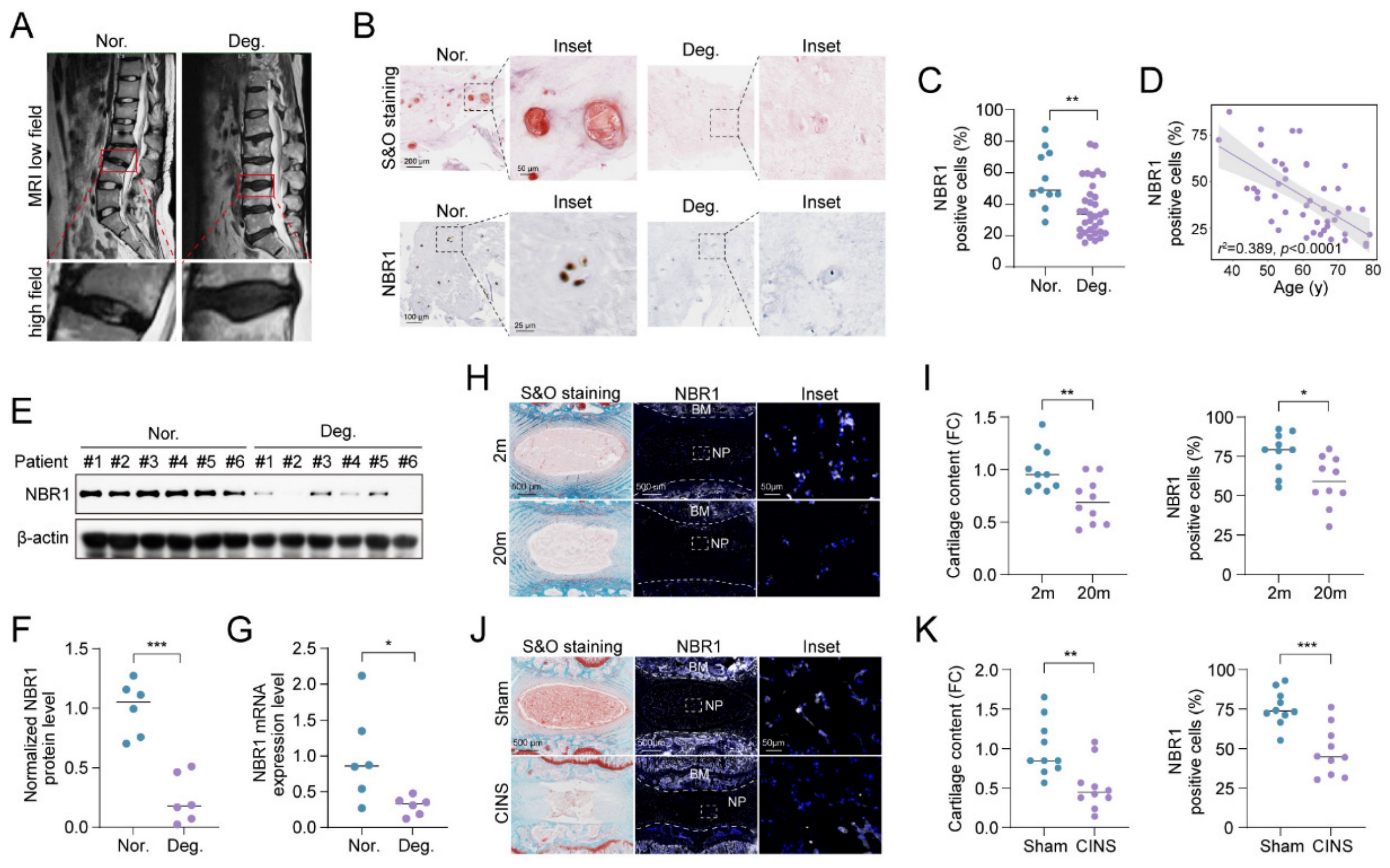
**NBR1 is downregulated in degenerative NPCs.** (**A**) Representative magnetic resonance T2-weighted images of normal (Nor.) and degenerated (Deg.) groups. The lower panels show pictures of the operated segments at a high magnification. (**B**) Representative images of human NP tissues stained with S&O (upper panel) or immunostained with NBR1 (lower panel). (**C**) Quantification of NBR1 positive cells in normal (n=11) and degenerated (n=34) groups. Data were analyzed using the Mann-Whitney U test and expressed as the means ± SEM. **P<0.01. (**D**) Linear regression analysis between NBR1 positive cell rate (%) and age of patients.**,** as determined by Western blot. (**E** and** F**) NBR1 protein expression in NP tissues isolated from patients in normal (n=6) or degenerated (n=6) group. (**G**) NBR1 mRNA expression in NP tissues isolated from patients in normal (n=6) or degenerated (n=6) group, as determined by qRT-PCR. (**H**) Representative images of intervertebral disc segments from rats aged 2 months or 20 months, sections were stained with S&O (left panel), or immunostained with NBR1 (middle and right panel). (**I**) Quantification of Safranin red intensity (left panel) and NBR1 positive cells (right panel) in NP tissues obtained from 2-month-old (n=10) or 20-month-old rats (n=10). FC: fold change. (**J**) Representative images of intervertebral disc segments from rats in sham or CINS group. (**K**) Quantification of Safranin red intensity (left panel) and NBR1-positive cells rate (right panel) in NP tissues obtained from rats in sham or CINS group. Statistical data were analyzed using two-tailed Student's t test and expressed as the means ± SEM. *P<0.05, **P<0.01, ***P<0.001.

To elucidate the role of NBR1 in IDD, we examined the expression of NBR1 in intervertebral disc samples from IDD patients. The classification of these tissues was determined based on preoperative MRI T2-weighted images, utilizing the Pfirrmann grading system^21^. As shown in Figure 1A, NP tissues with a Pfirrmann grade <3 were included in the normal group, while those with a Pfirrmann grade ≥3 were included in the degeneration group. Safranin O (S&O) staining exhibited a conspicuous decline in proteoglycan content within the tissues of degeneration group (Figures 1B and S1A). Immunohistochemical (IHC) staining demonstrated a significant reduction in the quantity of NBR1 positive cells within the degenerated NP tissues compared to the normal group (Figures 1B, C). Additionally, it was observed that NBR1 expression displayed a negative correlation with the age of patients (Figure 1D). We also found that both the expression of protein and mRNA levels were decreased in degenerated human NP tissues (Figures 1E-G). Interestingly, this decrease in NBR1 expression was not observed in annulus fibrosus cells (AFCs), which are characterized by a circular arrangement of the matrix (Figures S1B, C). Additionally, the rat coccygeal IDDs needle stab (CINS) and natural aging models were established to simulate the injury-induced or senescence-induced IDD. The degenerative degree of rat intervertebral disc was evaluated utilizing S&O staining (Figures 1H, I). Consistent with the findings observed in human samples, IF staining demonstrated that NBR1 expression was drastically decreased within the NP tissues of rat obtained from 20-month-old rats compared to those from 2-month-old rats (Figures 1H, I). Besides, the reduction of NBR1 expression was also observed in NP tissues of the CINS model (Figures 1J, K). However, no changes in NBR1 expressions were observed in rat AFCs from either the CINS or natural aging model (Figures S1D-G).

### 3.2 NBR1 retards senescent phenotypes of NPCs in IDD

**Figure 2:**
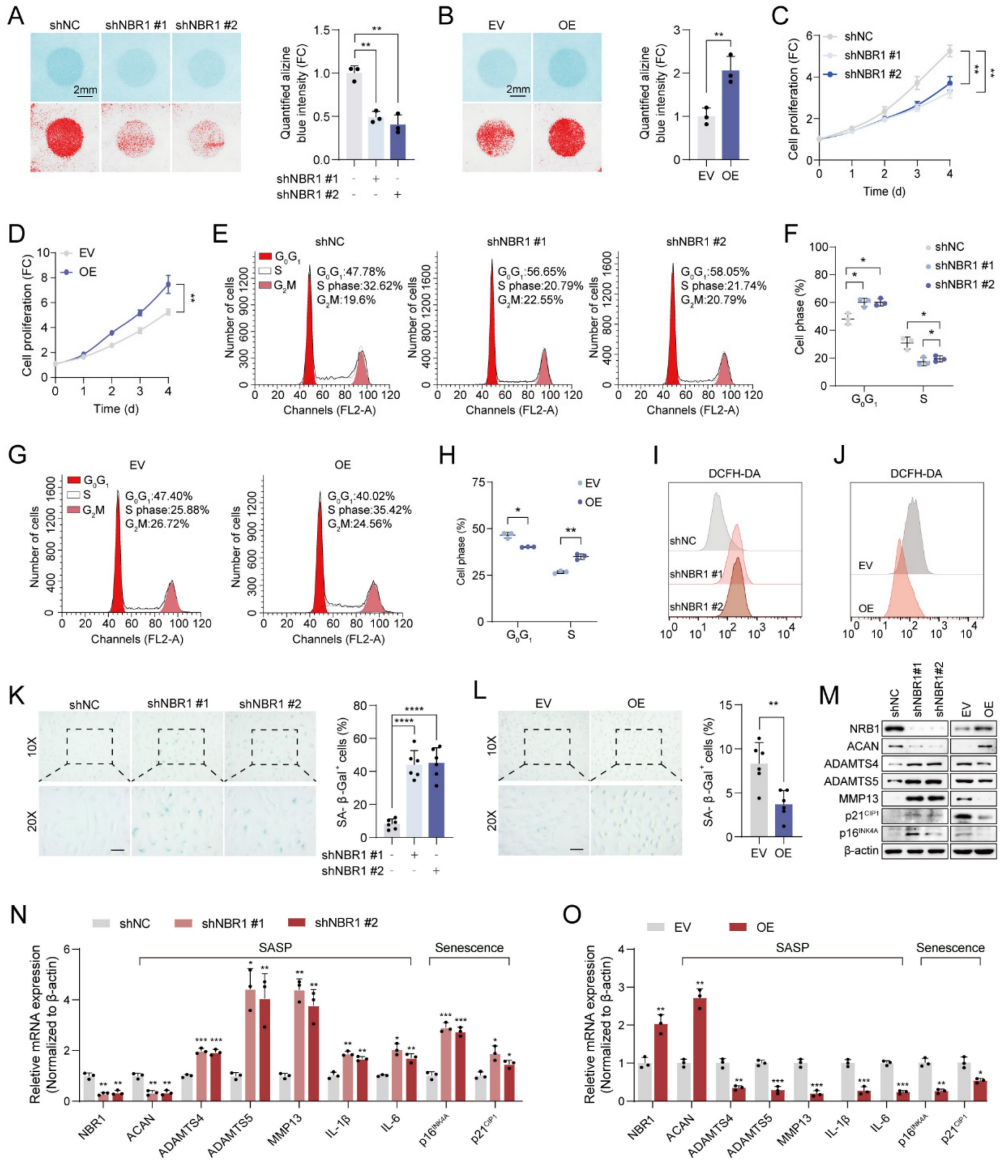
** NBR1 regulates NPCs ECM metabolism and senescent phenotypes.** (**A**) Representative Alcian blue staining (upper panel) and corresponding shifted images (lower panel) of NPCs infected with shNC or shNBR1 lentivirus. (**B**) Representative Alcian blue staining (upper panel), corresponding shifted images (lower panel) and quantification (right panel) of NPCs infected with empty vector (EV) or NBR1 overexpression (OE) lentivirus. Growth curves of NBR1 silence (**C**) or overexpression (**D**) NPCs. (**E** and **F**) Flow cytometry analysis for cell cycle distribution of NPCs infected with shNC or shNBR1 lentivirus. (**G** and **H**) Flow cytometry analysis and quantification for cell cycle distribution of NPCs infected with empty vector or NBR1 overexpression lentivirus. Flow cytometry analysis for DCFH-DA staining of NBR1 silence (**I**) or overexpression (**J**) NPCs. (**K**) Representative images (left panel) of SA-β-gal staining and quantification (right panel) of NPCs infected with shNC or shNBR1 lentivirus. Scale bar: 100 μm. (**L**) Representative images (left panel) and quantification (right panel) of SA-β-gal staining in NPCs infected with empty vector or NBR1 overexpression lentivirus. (**M**) NBR1, ACAN, ADAMTS4, ADAMTS5, MMP13, p21^CIP1^, p16^INK4A^ protein levels in NBR1 silence (left panel) or overexpression (right panel) NPCs, as determined by Western blot. NBR1, ACAN, ADAMTS4, ADAMTS5, MMP13, IL-1β, IL-6, p16^INK4A^, p21^CIP1^ in NBR1 silence (**N**) or overexpression (**O**) NPCs, as determined by qRT-PCR. Statistical data were analyzed using two-tailed Student's t test and expressed as the means ± SEM. *P<0.05, **P<0.01, ***P<0.001, ****P<0.0001.

To elucidate the biological function of NBR1 in IDD, we employed shNBR1 lentivirus or overexpression lentivirus to infect NPCs. The ECM secreted by NPCs plays a crucial role in maintaining intervertebral disc homeostasis^4^. Alcian blue staining revealed that NBR1 knockdown accelerated the ECM catabolism, whereas NBR1 overexpression promoted the anabolism (Figures 2A, B). Subsequently, the impact of NBR1 on the viability of NPCs was assessed through a CCK8 assay. It was observed that NBR1 knockdown hindered NPC proliferation, while NBR1 overexpression enhanced NPC viability (Figures 2C, D). To gain further insight into the mechanisms underlying NBR1's promotion of cell proliferation, flow cytometry was employed to investigate the impact of NBR1 alteration on cell cycle progression, and cell apoptosis. The flow cytometry analysis demonstrated that the silencing of NBR1 resulted in the arrest of the cell cycle at the *G0/G1* phase, as depicted in Figures 2E and F. Conversely, the overexpression of NBR1 accelerated the transition of the cell cycle from *G0/G1* to *S* phase in NPCs (Figures 2G, H). However, neither the knockdown nor the overexpression of NBR1 had impact on the NPCs apoptosis (Figures S2A-D). Additionally, the staining of DCFH-DA revealed that knockdown of NBR1 significantly elevated the intracellular level of ROS, while the overexpression of NBR1 had the opposite effect (Figures 2I, J and Extended Figures S2E, F). Cell cycle arrest, elevated ECM catabolism and intracellular ROS level are hallmarks of cellular senescence^25^. Therefore, we wondered whether NBR1 inhibits intervertebral disc degeneration by retarding cellular senescence of NPCs. SA-β-gal staining revealed a significant increase in the number of senescent cells upon NBR1 knockdown, while NBR1 overexpression decreased the cellular senescence of NPCs. Furthermore, Western blotting and qRT-PCR assays demonstrated that NBR1 knockdown promoted the expression of ECM anabolism-related gene ACAN and suppressed the expression of ECM catabolism-related genes ADAMTS4, ADAMTS5, and MMP13. Additionally, NBR1 knockdown also enhanced the expression of SASP-related cytokines IL-1β and IL-6, as well as senescence-related markers p16^INK4A^ and p21^CIP1^ (Figures 2M, N). Conversely, the overexpression of NBR1 exhibited the opposite effect (Figures 2M, O). Collectively, these results suggested that NBR1 plays a role in inhibiting the cellular senescence of NPCs in IDD.

### 3.3 NBR1 regulates senescent phenotypes via its selective autophagy-dependent function

To understand the fundamental mechanisms involved in the degeneration of NPCs mediated by NBR1, we first examined the autophagic level in NBR1-silenced NPCs. As shown in Figure S2G, the autophagic flux, as indicated by LC3B/A, remained unchanged following NBR1 silencing in NPCs, regardless of the presence or absence of the autophagy inhibitor, Baf-A1, thereby aligning with prior research findings^18^.

**Figure 3:**
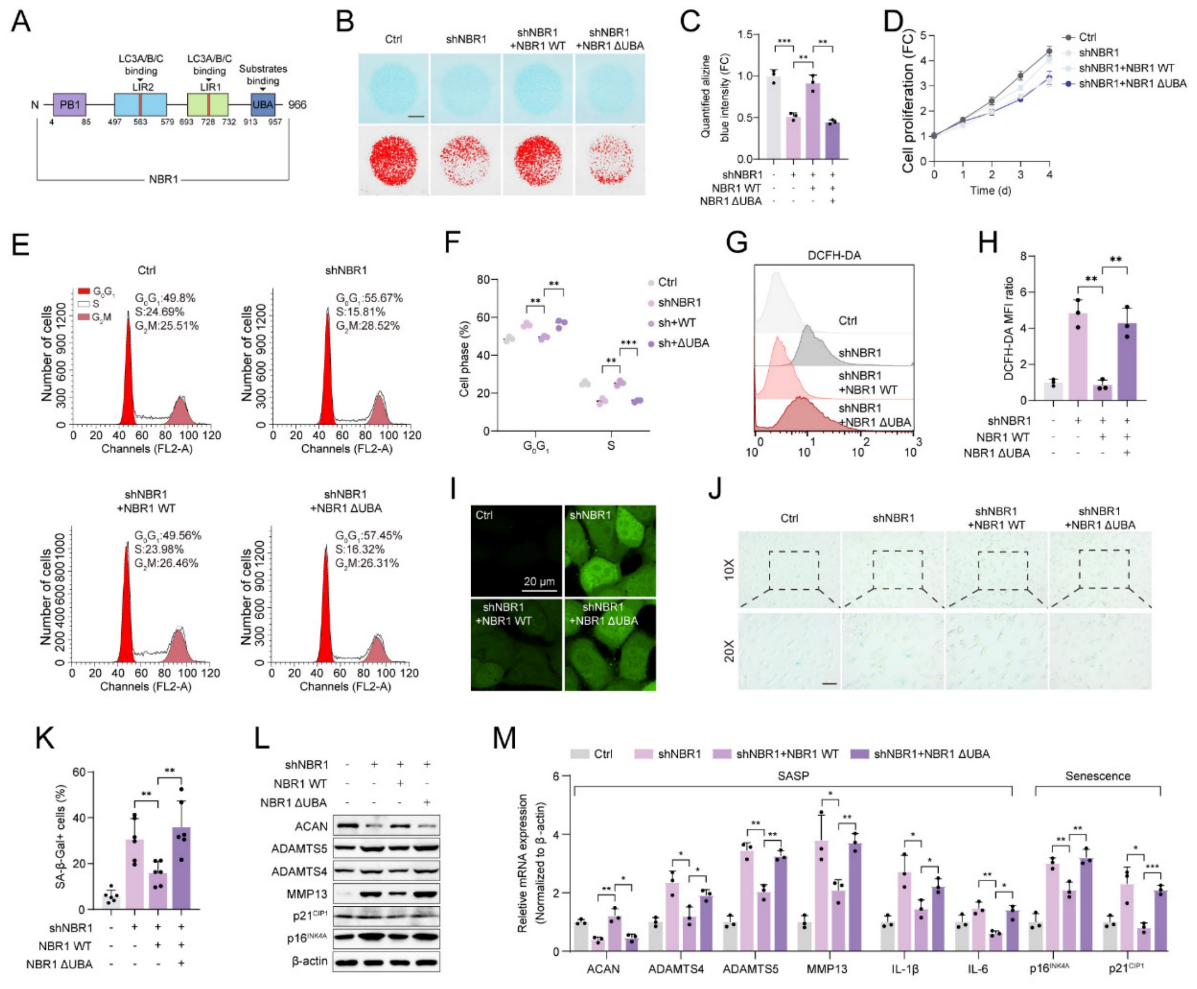
** NBR1 mediates NPCs ECM metabolism and senescent phenotypes via its autophagy-dependent function.** (**A**) Schematic illustration of full-length NBR1. (**B** and** C**) Representative Alcian blue staining (upper panel) and corresponding shifted images (lower panel) of NPCs infected with shNC, shNBR1, shNBR1+NBR1 WT, or shNBR1+NBR1 ΔUBA lentivirus. (**D**) Growth curve of NPCs in various experimental groups. (**E** and **F**) Flow cytometry analysis for cell cycle distribution of NPCs in various experimental groups. (**G** and **H**) Flow cytometry analysis for DCFH-DA staining of NPCs in various experimental groups. (**I**) Representative confocal images of DCFH-DA staining in various experimental groups. (**J** and** K**) Representative images and quantification of SA-β-gal staining of NPCs in various experimental groups. Scale bar: 100 μm. (**L**) ACAN, ADAMTS4, ADAMTS5, MMP13, p21^CIP1^, p16^INK4A^ protein levels of NPCs in various experimental groups. (**M**) ACAN, ADAMTS4, ADAMTS5, MMP13, IL-1β, IL-6, p16^INK4A^ and p21^CIP1^ mRNA levels in various experimental groups. Statistical data were analyzed using two-tailed Student's t test and expressed as the means ± SEM. *P<0.05, **P<0.01, ***P<0.001.

This suggests that NBR1's involvement in degeneration of NPCs operates autonomously, without interaction with non-selective autophagy. NBR1 is a multidomain scaffold protein which has been found to potentially have both autophagy-dependent and -independent functions (Figure 3A)^19^, ^20^. To clarify whether NBR1-mediated degeneration phenotype is associated with its role in selective autophagy, In this study, we generated a mutant NBR1 lacking the ubiquitin-associated (UBA) domain, which is known to be essential for the identification and degradation of ubiquitination substrates^16^. We then assessed the impact of this mutant on senescent phenotypes of NPCs. Alcian staining revealed that overexpression of NBR1 WT, but not the UBA-mutant, rescued the ECM catabolism caused by NBR1 knockdown (Figures 3B, C). Similarly, the overexpression of NBR1-ΔUBA failed to reverse the inhibitory effect on cell proliferation caused by NBR1 knockdown (Figure 3D). Flow cytometry analysis demonstrated that NBR1-ΔUBA was unable to rescue the *G0/G*1 arrest of NPCs, whereas WT NBR1 was capable of doing so (Figures 3E, F). Additionally, the elevated intracellular ROS level caused by NBR1 knockdown, was attenuated by the overexpression of NBR1 WT, but not NBR1-ΔUBA (Figures 3G-I). Importantly, the increase in SA-β-gal positive NPCs resulting from NBR1 knockdown was attenuated by the overexpression of NBR1 WT, but not NBR1-ΔUBA. Finally, we confirmed this effect using WB and qRT-PCR assays. As shown in Figure 3L, M. the expressions of SASP and senescence-related genes were restored by the overexpression of WT NBR1, but not NBR1-ΔUBA. In conclusion, these findings suggest that NBR1 plays a role in inhibiting the degeneration of NPCs through its ubiquitin-dependent selective autophagy mechanism.

### 3.4 4D label-free quantitative proteomic analysis revealed a senescence landscape in NBR1 knockdown NPCs

**Figure 4:**
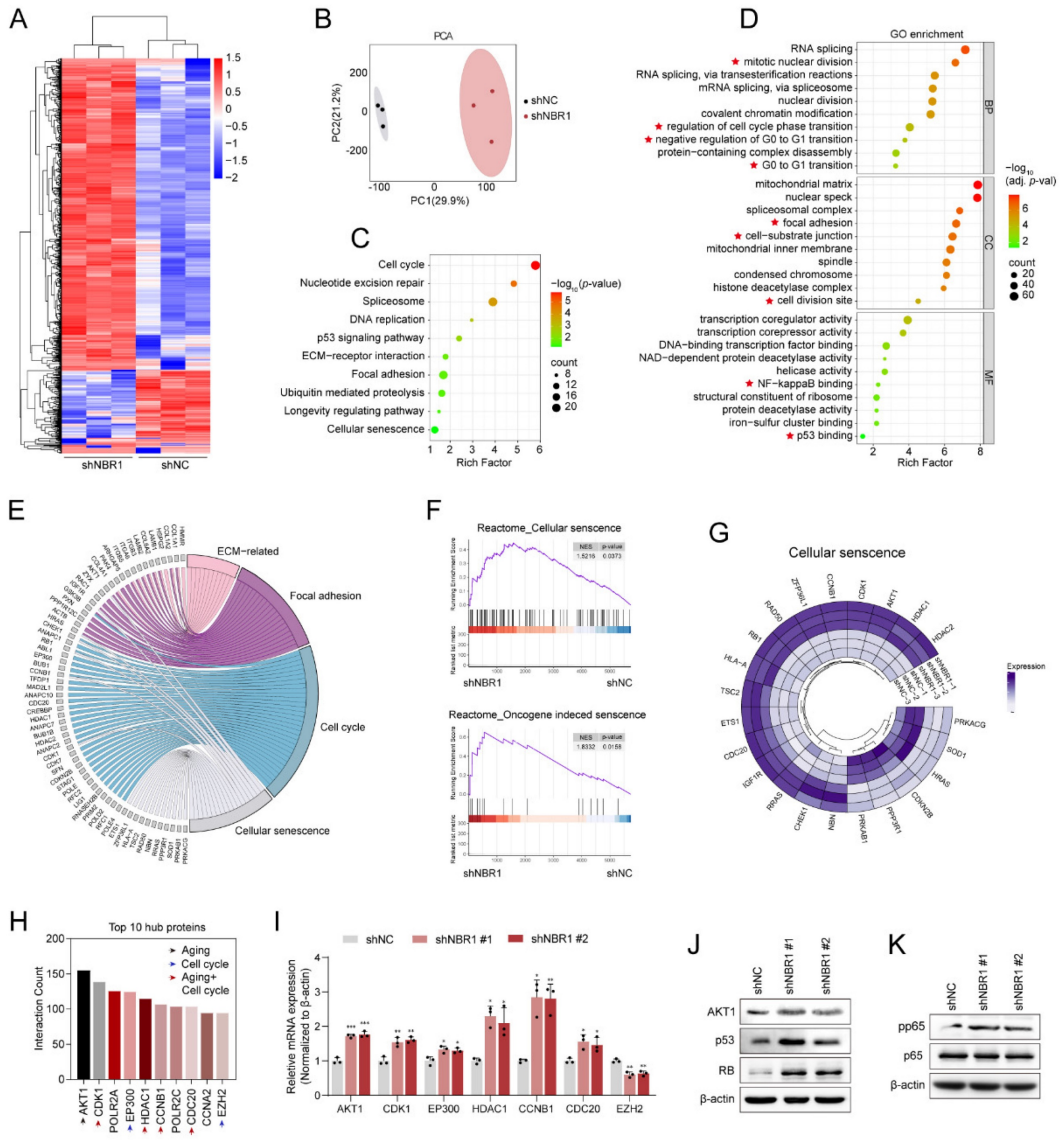
** NBR1 knockdown revealed a senescent landscape in NPCs.** (**A**) Hierarchical clustering analysis for differentially expressed proteins. (**B**) Principal component analysis (PCA) of the full protein profile of NPCs infected with shNC or shNBR1. (**C**) KEGG enrichment analysis of differentially expressed proteins. (**D**) GO analysis bar plot of biological process, cellular component, and molecular function. (**E**) Crossover of proteins in ECM-related, focal adhesion, cell cycle, and cellular senescence pathways. (**F**) GSEA analysis of cellular senescence and oncogene induced senescence based on the proteomic profiles. (**G**) Circos plot comparing differentially expressed proteins in NPCs after transfected with shNBR1 lentivirus, highlighting proteins related to cellular senescence. (**H**) Top 10 proteins with highest network connectivity. (**I**) AKT1, CDK1, EP300, HDAC1, CCNB1, CDC20. EZH2 mRNA levels in NPCs infected with shNC or shNBR1 lentivirus, as determined by qRT-PCR. (**J**) AKT1, p53 and RB protein levels in NBR1 silence NPCs. (**K**) pp65 and p65 protein levels in NBR1 silence NPCs. Statistical data were analyzed using two-tailed Student's t test and expressed as the means ± SEM. *P<0.05, **P<0.01, ***P<0.001.

In order to elucidate the mechanism by which NBR1 acts on NPCs, we conducted a 4D Label-free proteomic analysis after infecting NPCs with NBR1 shRNA or shNC lentivirus. As shown in the heatmap (Figure 4A), a total of 1082 differentially expressed proteins were identified, with the majority being upregulated (819 proteins) and 263 downregulated, thereby indicating the crucial regulatory function of NBR1 in preserving intracellular protein stability. The results of the PCA analysis indicated that the NPCs with NBR1 knockdown exhibited a distinct proteome shift in comparison to the shNC group (Figure 4B). The KEGG analysis revealed a significant enrichment in pathways related to ECM interaction, focal adhesion, cell cycle, and cellular senescence. Furthermore, the GO analysis demonstrated a notable enrichment in biological process terms associated with the cell cycle, particularly the *G0/G1* transition, which aligns with our *in vitro* findings (Figure 2G, H). In terms of cellular component (CC) term, there was observed enrichment in pathways related to the ECM, including cell-substrate junctions and cell division sites. Additionally, the molecular function (MF) term exhibited enrichment in NF-κβ and p53 binding pathways (Figure 4D). Based on the results obtained from the KEGG enrichment analysis, we have compiled an extensive inventory of differentially expressed proteins that are linked to ECM, focal adhesion, cell cycle, and cell senescence (Figure 4E). Additionally, the GSEA analysis has revealed a noteworthy upregulation of pathways associated with cellular senescence and oncogene-induced senescence in the NBR1 knockdown group (Figure 4F). The expression profiles of proteins related to cellular senescence and cell cycle were presented in Figure 4G and Figure S3A. Furthermore, PPI network analysis was performed to identify crucial proteins in response to NBR1 knockdown. Proteins with a higher degree of interaction were regarded as potential hub proteins^26^. The top 10 hub proteins were presented in Figure 4H, with a majority (7 out of 10) being linked to cellular senescence or cell cycle pathways. Additionally, the mRNA expression levels of these hub proteins except for EZH2 were confirmed to be significantly increased following NBR1 knockdown (Figure 4I). According to reports, AKT1, a protein that exhibits the highest degree of interaction among differentially expressed proteins, has the ability to induce cellular senescence and arrest the cell cycle in the *G0/G1* phase by activating the p53/p21 and p16/RB pathways^27^, ^28^. Western blot analysis revealed an upregulation in protein levels of AKT1, p53, and RB in NBR1-silenced NPCs (Figure 4J). Furthermore, p-p65 NF-κβ, a known regulator of SASP^29^, showed an increased expression in NBR1-silenced NPCs (Figure 4K). Consequently, these findings provide compelling evidence that NBR1 knockdown shapes the senescence landscape in NPCs.

### 3.5 NBR1 selectively targets and clears SRBD1 in NPCs through the autophagic-lysosomal pathway

**Figure 5:**
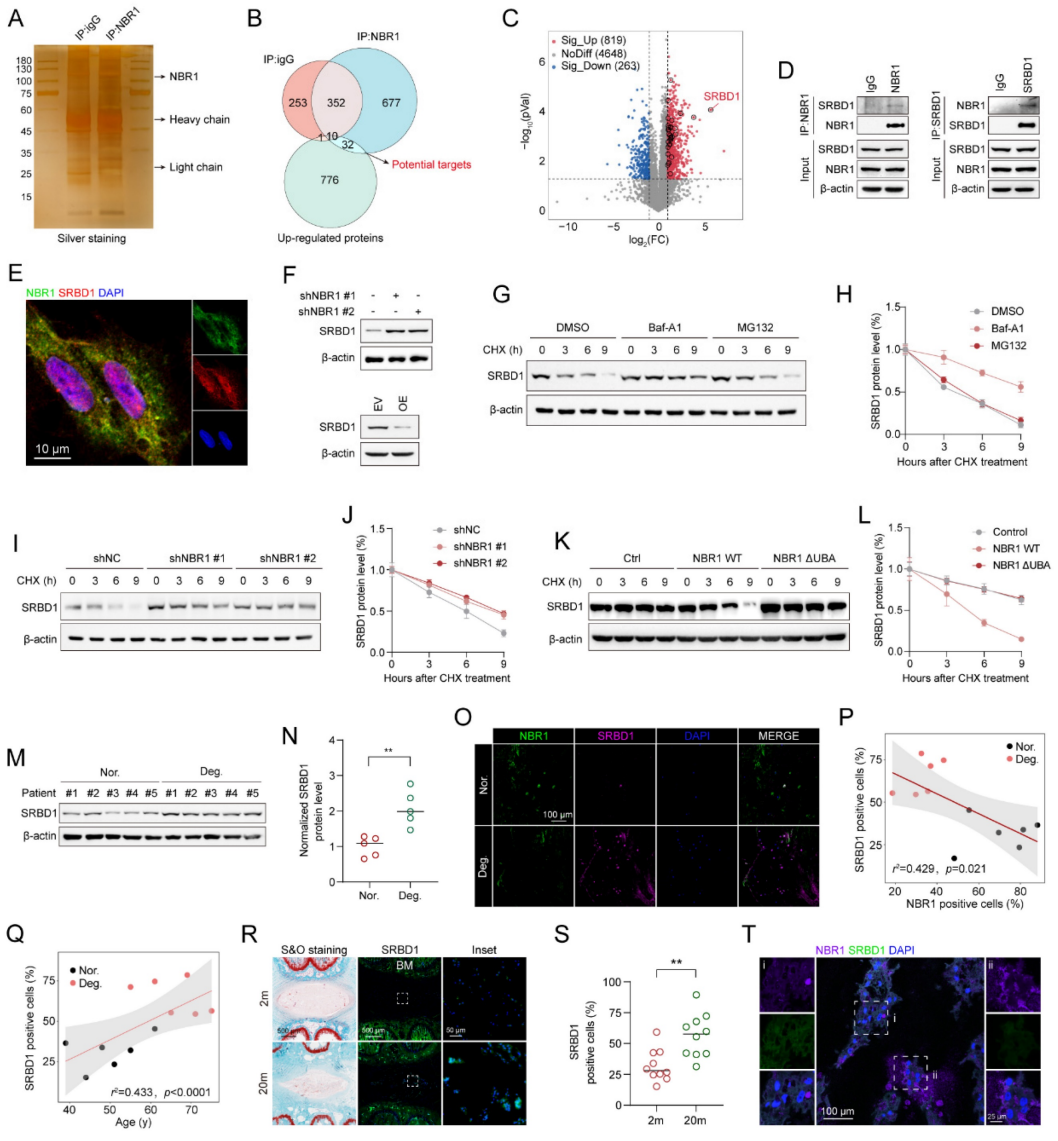
** NBR1 selectively targets and clears SRBD1 in NPCs through the autophagic-lysosomal pathway.** (**A**) Silver staining images of proteins immunoprecipitated with IgG or NBR1 antibodies. (**B**) Venn diagram showing the overlaps between anti-IgG, anti-NBR1 conjugated proteins and upregulated proteins in proteomic analysis. (**C**) Volcano plots of differentially expressed proteins. Potential targets of NBR1 were marked with black circles. (**D**) Immunoblot results of proteins immunoprecipitated with IgG, NBR1, SRBD1 antibodies. (**E**) Confocal images revealed the localization of NBR1 and SRBD1 in NPCs. (**F**) SRBD1 protein levels in NBR1 silence (upper panel) or overexpression (lower panel) NPCs. (**G** and **H**) The half-life of SRBD1 in NPCs treated with CHX (25 μg/mL, 3 h intervals) plus Baf-A1 (4 nM) or MG132 (20 μM). (**I** and **J**) The half-life of SRBD1 in NBR1 silence NPCs. (**K** and **L**) The half-life of SRBD1 in NBR1 silence NPCs with NBR1 WT or ΔUBA overexpression. (**M** and **N**) SRBD1 protein expression in NP tissues isolated from patients in normal (n=5) or degenerated (n=5) group, as determined by Western blot. (**O**) Representative images of human NP tissues immunostained with NBR1 and SRBD1 in normal (n=6) and degenerated group (n=6). (**P**) Linear regression analysis between NBR1 and SRBD1-positive cell rate (%) in normal (n=6) and degenerated group (n=6). (**Q**) Linear regression analysis between SRBD1 positive cell rate (%) and age of patients. (**R**) Representative images of intervertebral disc segments from rats aged 2 months or 20 months, sections were immunostained stained with NBR1 (middle and right panel). (**S**) Quantification of NBR1 positive cell rate (%) in NP tissues obtained from 2-month-old (n=10) or 20-month-old rats (n=10). (**T**) Immunostaining for NBR1 and SRBD1 in NP tissues from 20-month-old rats. The boxed regions indicate areas of high (**ii**) or low (**i**) NBR1 expression. Statistical data were analyzed using two-tailed Student's t test and expressed as the means ± SEM. *P<0.05, **P<0.01, ***P<0.001.

The aforementioned findings suggest that NBR1 mediates NPCs senescence through its autophagy-dependent function. However, the knockdown of NBR1 regulates the expression of hub proteins at the mRNA level (Figure 4I). In order to identify the specific downstream targets of NBR1, we conducted protein immunoprecipitation using NBR1 antibody, followed by mass spectrometry analysis (Figure 5A). Through the integration of proteomics and IP-MS data, we identified 32 proteins as potential direct downstream targets of NBR1 (Figure 5B), the expression profile of these proteins was presented in the volcano plot (Figure 5C), detail information was listed in **Table S3**. Among these proteins, S1 RNA-binding domain-containing protein 1 (SRBD1) exhibited a noteworthy upregulation of 50.6-fold subsequent to NBR1 knockdown. Previous investigation highlighted the crucial involvement of SRBD1 in the age-related disease, glaucoma^30^. Therefore, we chose SRBD1 as the subject for further investigation. Through immunoprecipitation and Western blot analysis, we confirmed the interaction between NBR1 and SRBD1 (Figure 5D). Additionally, the immunofluorescence result indicated that NBR1 is predominantly localized in the cytoplasm, whereas SRBD1 is distributed in both the nucleus and cytoplasm, their primary interaction occurs within the cytoplasm (Figure 5E). Furthermore, through the manipulation of NBR1 expression levels, we observed corresponding increases or decreases in the protein expression level of SRBD1 (Figure 5F). Importantly, the mRNA level of SRBD1 remained unchanged (Figures S3D). This suggests that NBR1 primarily governs the expression of SRBD1 by influencing the stability of SRBD1 protein. Furthermore, the depletion of two additional prominent selective autophagy receptors, namely SQSTM1 and TAX1BP1, did not elicit any alterations in SRBD1 (Figure S3B and C). Next, we sought to investigate the potential impact of NBR1-mediated autophagic lysosomal pathway on the intracellular stability of SRBD1. Immunoblotting results demonstrated that the utilization of Baf-A1 significantly mitigated the intracellular degradation of SRBD1. Conversely, the introduction of MG132, a proteasome inhibitor, exhibited minimal influence on the stability of SRBD1 (Figures 5G, H). Moreover, the depletion of NBR1 resulted in an augmented protein stability of SRBD1 (Figures 5I, J). Notably, overexpression of wild-type NBR1, rather than NBR1-ΔUBA in cells with NBR1 knockdown significantly accelerated the degradation of SRBD1 (Figures 5K, L). Taken together, these findings suggest that SRBD1 undergoes intracellular degradation via the autophagic lysosomal pathway mediated by NBR1.

Next, we examined the expression of SRBD1 in NPCs from IDD patients. a significant increase in SRBD1 protein level was observed in degenerated human NPCs compared to normal NPCs (Figures 5M, N), interestingly, with no significant change in its mRNA levels (Figure S3E). Immunofluorescence staining using NBR1 and SRBD1 antibodies in normal and degenerated human NP tissues revealed an upregulation of SRBD1 expression in degenerated NP tissues, which exhibited a negative correlation with NBR1 expression but a positive correlation with the age of patients (Figures 5P, Q). Additionally, compared to NP tissues from 2-month-old rats, the number of SRBD1-positive cells in the NP tissues from 20-month-old rats was significantly increased (Figures 5R, S). Notably, SRBD1 is downregulated in NBR1-expressing NPCs *in vivo* (Figure 5T). In summary, these findings collectively illustrate that NBR1 in NPCs selectively targets and clears SRBD1 through the autophagic-lysosomal pathway.

### 3.6 NBR1 regulates the senescent phenotype of NPCs through modulation of SRBD1

**Figure 6:**
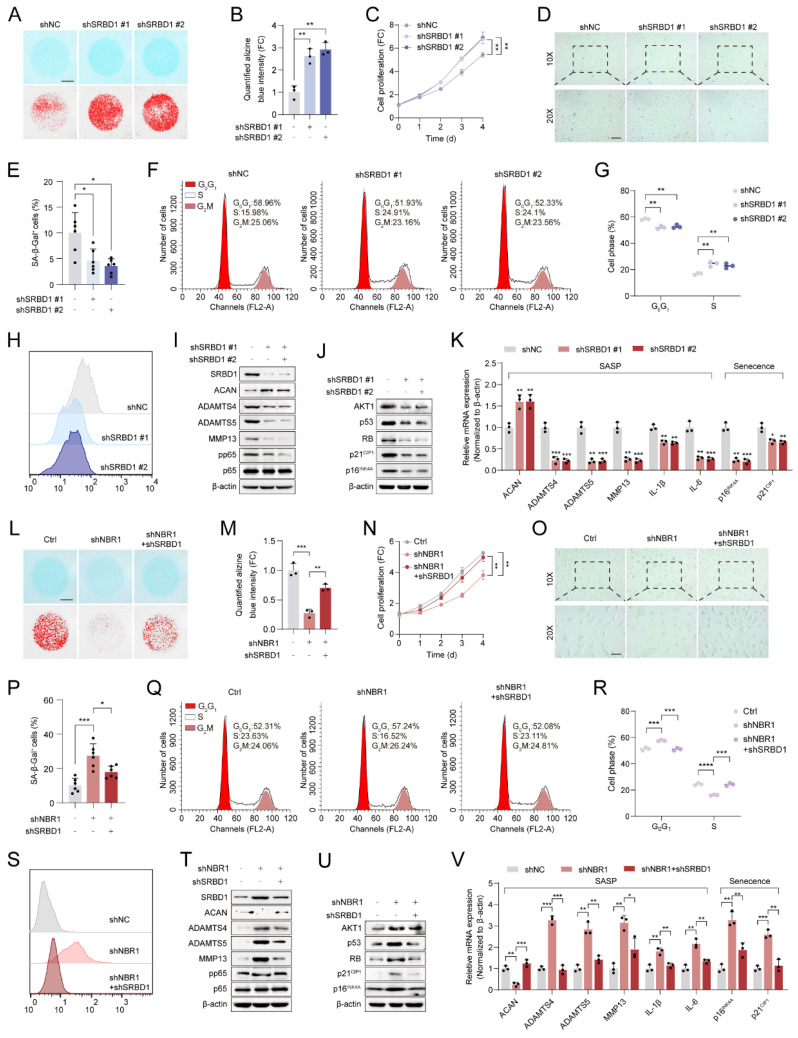
** NBR1 regulates the senescent phenotype of NPCs through modulation of SRBD1.** (**A**) Representative Alcian blue staining (upper panel) and corresponding shifted images (lower panel) of NPCs infected with shNC or shSRBD1 lentivirus. (**B**) Quantification of Alcian blue staining in (**A**). (**C**) Growth curve of NPCs infected with shNC or shSRBD1 lentivirus. (**D** and** E**) Representative images and quantification of SA-β-gal staining in NPCs infected with shNC or shSRBD1 lentivirus. Scale bar: 100 μm. (**F** and **G**) Flow cytometry analysis and quantification for cell cycle distribution of NPCs infected with shNC or shSRBD1 lentivirus. (**H**) Flow cytometry analysis for DCFH-DA staining of NPCs infected with shNC or shSRBD1 lentivirus. (**I**) SRBD1, ACAN, ADAMTS4, ADAMTS5, MMP13, pp65, p65 protein expression levels in SRBD1 silence NPCs, as determined by Western blot. (**J**) AKT1, p53, RB, p21^CIP1^, p16^INK4A^ protein expression levels in SRBD1 silence NPCs. (**K**) ACAN, ADAMTS4, ADAMTS5, MMP13, IL-1β, IL-6, p16^INK4A^ and p21^CIP1^ mRNA expression levels in SRBD1 silence NPCs, as determined by qRT-PCR. (**L**) Representative Alcian blue staining (upper panel) and corresponding shifted images (lower panel) of NPCs in various experimental group. (**M**) Quantification of Alcian blue staining in various experimental group. (**N**) Growth curve of NPCs in various experimental group. (**O** and **P**) Representative images and quantification of SA-β-gal staining of NPCs in various experimental groups. Scale bar: 100 μm. (**Q** and **R**) Flow cytometry analysis and quantification for cell cycle distribution of NPCs in various experimental groups. (**S**) Flow cytometry analysis for DCFH-DA staining of NPCs in various experimental groups. (**T**) SRBD1, ACAN, ADAMTS4, ADAMTS5, MMP13, pp65, p65 protein levels of NPCs in various experimental groups. (**U**) AKT1, p53, RB, p21^CIP1^, p16^INK4A^ protein levels of NPCs in various experimental groups. (**V**) ACAN, ADAMTS4, ADAMTS5, MMP13, IL-1β, IL-6, p16^INK4A^ and p21^CIP1^ mRNA expression levels in various experimental groups. Statistical data were analyzed using two-tailed Student's t test and expressed as the means ± SEM. *P<0.05, **P<0.01, ***P<0.001, ****P<0.0001.

Subsequently, the role of SRBD1 in senescent phenotypes in NPCs was assessed by infecting NPCs with shSRBD1 lentivirus. The results of Alcian blue staining indicated that the suppression of SRBD1 led to an increase in ECM secretion by NPCs (Figures 6A, B). The proliferation viability of NPCs was enhanced by SRBD1 knockdown, as revealed by the CCK8 assay (Figure 6C). Additionally, SA-β-gel staining revealed a reduction in the number of senescent cells (Figures 6D and E). Flow cytometry results showed an accelerated transition of the cell cycle from *G0/G1* to *S* phase (Figures 6F, G), along with a decrease in intracellular levels of ROS (Figure 6H and Figure S4A). The experimental findings from Western blotting and qRT-PCR further confirmed that the SRBD1 knockdown impeded the expression of markers associated with SASP and cellular senescence (Figures 6I-K).

To investigate the role of SRBD1 in the senescence process induced by NBR1 depletion, a rescue assay was conducted. In brief, SRBD1 was suppressed in NBR1 knockdown NPCs. The suppression of SRBD1 effectively counteracted various cellular effects caused by NBR1 knockdown, including ECM degradation (Figures 6L, M), inhibition of cell proliferation (Figure 6N), induction of cellular senescence (Figures 6O, P), cell cycle arrest (Figures 6Q, R), and elevated levels of ROS (Figure 6S and Figure S4B). Additionally, the protein and mRNA levels of markers associated with SASP and cellular senescence were also restored upon SRBD1 knockdown (Figures 6T-V). Taken together, these findings suggest that the induction of senescence in NPCs by NBR1 knockdown is mediated by SRBD1.

### 3.7 NBR1/SRBD1 axis regulates senescence of NPCs and IDD progression *in vivo*

**Figure 7:**
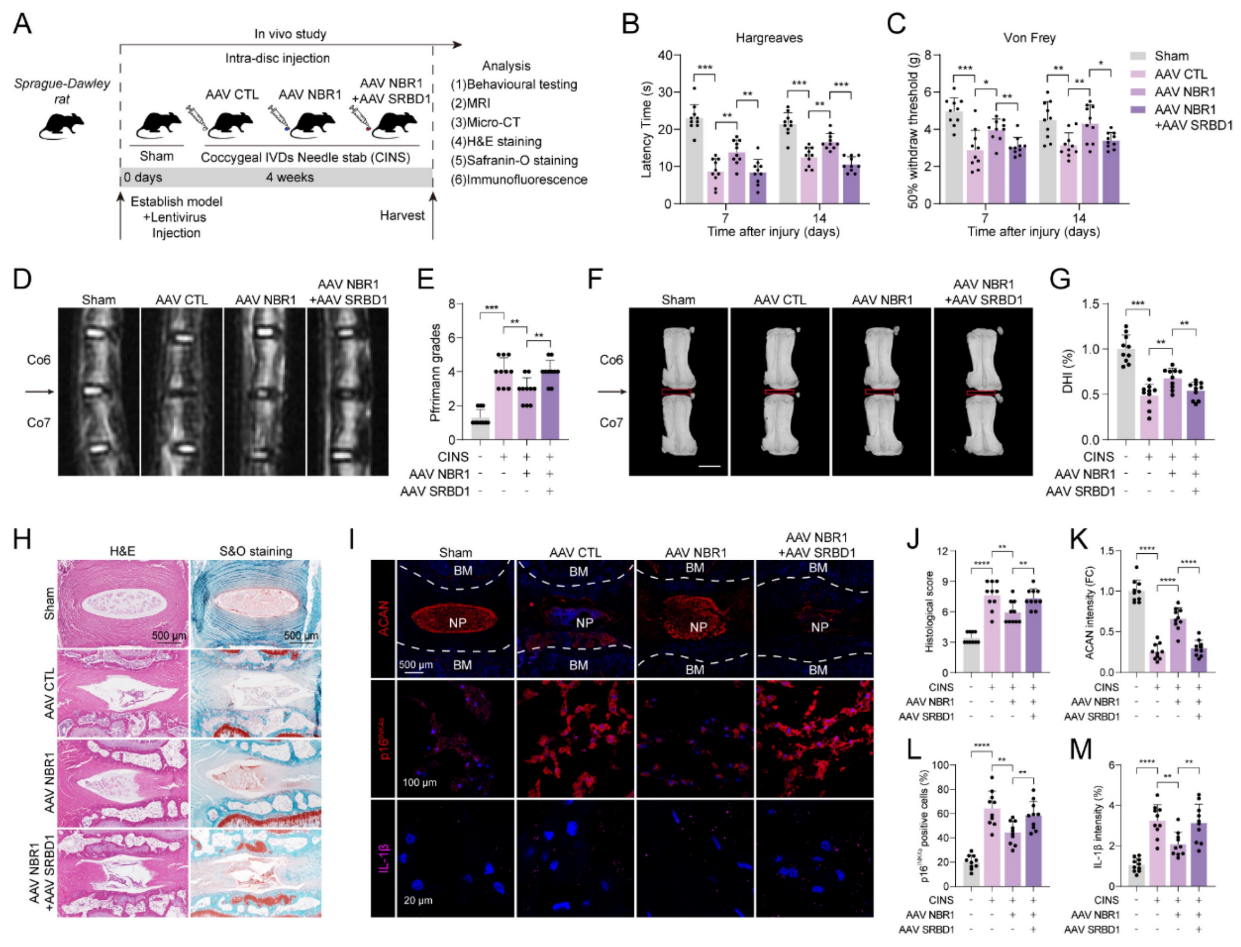
**NBR1/SRBD1 axis regulates NPCs senescence and IDD progression *in vivo*.** (**A**) Schematic illustration of CINS model establishment and experiment design to evaluate the effects of NBR1 and SRBD1 *in vivo*. (**B**) Hargreaves test was performed to evaluate the thermal hyperalgesia of rats in various experimental groups. (**C**) Von Frey test was performed to evaluate the mechanical allodynia of rats in various experimental groups. (**D** and** E**) Representative images of T2-weighted MRI and relative Pfirrmann grade of rat coccygeal after 4 weeks AAVs intradiscal injection. The arrow indicates the puncture segment. (**F** and **G**) Representative μCT images and quantified change levels of disc height index (DHI). (**H**) Representative images of rat coccygeal sections stained with H&E (left panel) or S&O (right panel) in various experimental groups. (**I**) Representative images of rat coccygeal sections immunostained with ACAN, p16^INK4A^, and IL-1β in various experimental groups. (**J**) Histological score of rat NP tissues obtained from various experimental groups according to S&O staining. (**K** and** L**) Quantification of ACAN and p16^INK4A^ positive cells in NP tissues obtained from various experimental groups. (**M**) Quantification of IL-1β intensity of NP tissues in various experimental groups. Statistical data were analyzed using two-tailed Student's t test and expressed as the means ± SEM. *P<0.05, **P<0.01, ***P<0.001, ****P<0.0001.

In order to verify the role of the NBR1/SRBD1 axis in IDD, we established a rat CINS model and introduced NBR1 or SRBD1 adeno-associated viruses (AAVs) into NPCs through intradiscal injection (Figure 7A). The effectiveness of the viral infection was assessed by immunofluorescence staining of SRBD1 (Figure S5A). One prominent clinical manifestation of intervertebral disc degeneration is the development of pain phenotypes^31^. Behavioral experiments demonstrated that rats in the control AAV group exhibited significantly increased thermal and mechanical hyperalgesia on postoperative days 7 and 14. In contrast, the administration of NBR1 AAV resulted in a significant reduction in pain perception in the rats. Notably, the co-administration of NBR1 and SRBD1 AAVs counteracted the pain-relieving effects of NBR1 overexpression (Figures 7B, C). The findings from magnetic resonance imaging (MRI) of the rats' disc demonstrated that the control group AAV exhibited pronounced dehydration in the nucleus pulposus region and a decrease in intervertebral disc height, in comparison to the sham group. However, the overexpression of NBR1 AAV alleviated these symptoms, and this beneficial effect was impeded by simultaneous infection with NBR1 and SRBD1 AAVs (Figures 7D-J). Moreover, we employed histological staining techniques, specifically H&E, S&O, and ACAN immunofluorescence staining, on intervertebral disc sections obtained from separate groups of rats. The results demonstrated that the introduction of NBR1 lentivirus led to an increase in proteoglycan content and a decrease in degeneration grade. Conversely, the simultaneous overexpression of NBR1 and SRBD1 demonstrated the capability to counteract this effect (Figures 7H-K). A recent study has revealed that acute injury can induce cellular senescence *in vivo*^32^. By employing p16^INK4A^ and IL-1β immunofluorescence staining, a significant reduction in senescent cells and IL-1β secretion was observed in the NP tissues of rats injected with the NBR1 AAV, in comparison to the control AAV group. Additionally, the simultaneous overexpression of NBR1 and SRBD1 AAV was found to reverse this effect (Figures 7I-M). In summary, these findings suggest that NBR1 plays a role in inhibiting the senescent phenotype of NPCs and delaying the progression of IDD by regulating SRBD1.

## 4. Discussion

**Figure 8:**
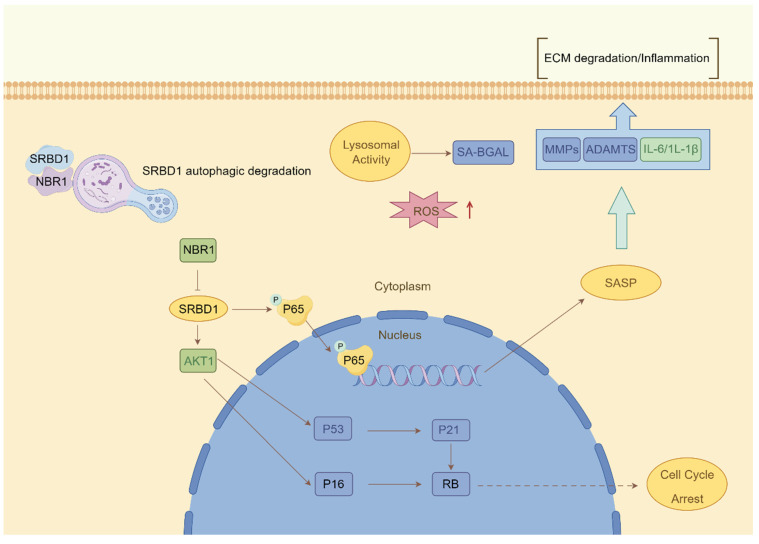
Schematic illustration describing the molecular mechanism by which NBR1 retards cellular senescence by degrading SRBD1.

Despite the ample evidence supporting the crucial role of autophagy in IDD, prior investigations have primarily concentrated on the impact of macroautophagy or non-selective autophagy on IDD. The precise involvement of selective autophagy in the regulation of IDD has remained uncertain. In this study, we present the preliminary evidence demonstrating the depletion of the selective autophagy receptor NBR1 in NPCs during the degeneration process. This depletion leads to the buildup of SRBD1 within NPCs, thereby accelerating NPCs senescence and initiating IDD (Figure 8). The strategy based on the NBR1/SRBD1 regulatory axis for targeted elimination of detrimental molecules presents a novel outlook for the management of IDD.

Autophagy, specifically non-selective autophagy, plays a vital role in regulating cellular homeostasis. During the process of IDD, non-selective autophagy seems to be activated in response to stressors such as nutrient deprivation and hypoxia^10^, ^33^. In this study, we report a decrease in the expression of the selective autophagy receptor NBR1 in degenerated NPCs, and this decrease is associated with aging. In the majority of age-related degenerative diseases, there is a prevalent occurrence of diminished autophagic flux as age advances^34^-^36^. This observation is not unexpected, as the decline in autophagic activity is indicative of cellular dysfunction, which is a prominent characteristic of the aging process^37^. Studies have demonstrated that the deletion of autophagy-related genes in mouse brain neurons can lead to the accumulation of impaired proteins, thereby contributing to the development of neurodegenerative disorders^38^, ^39^. Conversely, the manipulation of ATG5 expression via gene-editing methods in mice has been shown to substantially prolong lifespan of mice^40^. Interestingly, this decline appears to be observed specifically in the NPCs, this may be attributed to the harsher microenvironment that NPCs face during the degenerative process compared to AFCs. In conclusion, our research highlights the distinctive expression pattern of the selective autophagy receptor NBR1 in IDD.

The application of non-selective autophagy-related drugs with broad-spectrum activity in the treatment of IDD is hindered by various limitations. The non-selective nature of autophagy substrates often leads to the buildup of impaired molecules and organelles within vulnerable NPCs, potentially triggering autophagic cell death. This poses difficulties in integrating autophagy modulation into clinical therapies for age-related diseases^41^, ^42^. In contrast, NBR1, due to its distinctive substrate recognition pattern, primarily operates through selective autophagy-mediated elimination of specific substrates, thus exerting its influence on pathological conditions. For instance, in the context of viral infections, NBR1 plays a role in reducing the production of type I interferon by degrading IRF3 through autophagy, thereby assisting in the attenuation of the inflammatory response^43^. In pancreatic cancer cells, excessive autophagy facilitated by NBR1 contributes to immune evasion through the autophagic degradation of MHC-I. In the present study, we report that NBR1 knockdown significantly promotes senescent phenotypes in NPCs through its autophagy-dependent function^19^. Additionally, we identify a novel substrate of NBR1, SRBD1, and demonstrate that the loss of NBR1 in IDD leads to the accumulation of SRBD1, thereby mediating the senescent phenotype in NPCs.

Recent studies have highlighted the significant role of RNA-binding protein (RBP) in degenerative joint diseases^44^. The current body of research on SRBD1 primarily focuses on its association with glaucoma, an age-related disease^30^, ^45^. However, the molecular mechanisms underlying SRBD1's involvement in degenerative diseases and cellular senescence remain largely unexplored. Here, we propose that SRBD1 holds promise as a potential biomarker for senescence. It accumulates significantly in NPCs with impaired selective autophagy and mediates cellular senescence and SASP through p53/p21, p16/RB, and NF-κβ pathways.

Our study presents certain limitations. Firstly, the precise expression and mechanistic roles of additional proteins involved in selective autophagy, such as NDP52 and OPTN, n the context of IDD remain incompletely understood. Further investigation is necessary to unveil the intricate roles of selective autophagy mechanisms in the progression of IDD. Secondly, while this study has successfully identified SRBD1 as an aging biomarker, the specific mechanisms through which it influences cellular senescence are yet to be determined. Further clarification is required to ascertain the potential implication of its RNA-binding function.

In conclusion, our research provides innovative perspectives on the role of autophagy in IDD. Specifically, we have identified the absence of the selective autophagy receptor NBR1 in degenerated NPCs, leading to the induction of senescence in NPCs through the inhibition of autophagic degradation of SRBD1. Consequently, our findings suggest a potential therapeutic strategy for modulating NPCs senescence and IDD by targeting the NBR1/SRBD1 axis.

## Supplementary Material

Supplementary figures and tables.

## Abbreviations

IDD: intervertebral disc degeneration
NPCs: nucleus pulposus cells
AFCs: annulus fibrosus cells
SASP: senescence-associated secretory phenotype
ECM: extracellular matrix
ACAN: aggrecan
NBR1: neighbor of BRCA1
SAR: selective autophagy receptor
LIR: LC3 interacting regions
UBA: ubiquitin-associated
LC-MS: liquid chromatography-tandem mass spectrometry
CINS: coccygeal IDDs needle stab
IHC: immunohistochemistry
HE: Hematoxylin and eosin
S&O: Safranin O
AAV: adeno-associated viral vector
