# Supplementary material for: Selective Autophagy Receptor NBR1 Retards Nucleus Pulposus Cell Senescence by Directing the Clearance of SRBD1
Source: Int J Biol Sci. 2024 Jan 1;20(2):701–17. doi: 10.7150/ijbs.90186 (PMC10758090; doi:10.7150/ijbs.90186)
Supplement: Supplementary file 1 — Supplementary figures and tables. [file ijbsv20p0701s1.pdf]

Supplementary materials

Selective autophagy receptor NBR1 retards nucleus pulposus cell senescence by directing the clearance of SRBD1

Honghai Song[1, 2, †], Yutao Zhu[1, 2, †], Chuan Hu[1, 2, †], Qianyu Liu[3], Yang Jin[1, 2], Pan Tang[1, 6], Jiechao Xia[1, 2], Dingqi Xie[1, 2], Sicheng Jiang[1, 2], Geliang Yao[4, 5], Zhili Liu[4, 5 #], Zhijun Hu[1, 2, #].

[1] Department of Orthopaedic Surgery, Sir Run Run Shaw Hospital, Medical College of Zhejiang University, Hangzhou 310016, China

[2] Key Laboratory of Musculoskeletal System Degeneration and Regeneration Translational Research of Zhejiang Province, Hangzhou 310016, China

[3] Ministry of Education Key Laboratory of Biosystems Homeostasis & Protection, College of Life Sciences, Zhejiang University, Hangzhou 310058, China

[4] Department of Orthopedic Surgery, The First Affiliated Hospital of Nanchang University, Nanchang 330006, China

[5] Institute of Spine and Spinal Cord, Nanchang University, Nanchang 330006, China

[6] Department of Orthopaedics, Affiliated Huzhou Hospital, Zhejiang University School of Medicine, Huzhou 313000, China

Supplementary Figures

Supplementary Fig. S1

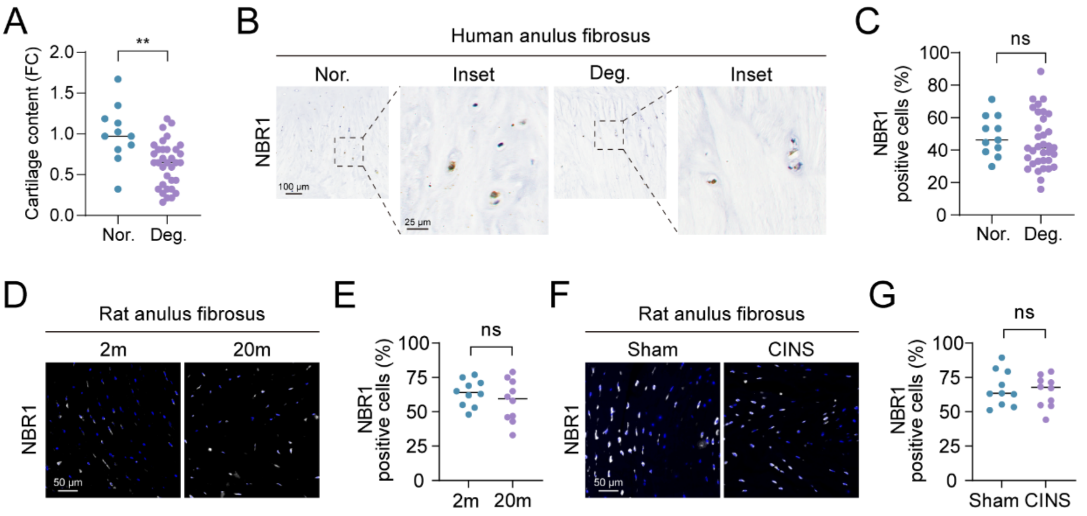

Supplementary Fig. S1 NBR1 is specifically downregulated in NPCs. (**A**) Quantification of S&O staining (red) in normal (n=11) and degenerated (n=34) groups. (**B** and **C**) Representative images and quantification of human annulus fibrosus tissues from normal (n=11) or degenerated group. (**D** and **E**) Representative images of annulus fibrosus from rats aged 2 months or 20 months, sections were immunostained with NBR1. (**F** and **G**) Representative images of annulus fibrosus from rats in sham or CINS group, sections were immunostained with NBR1.

Supplementary Fig. S2

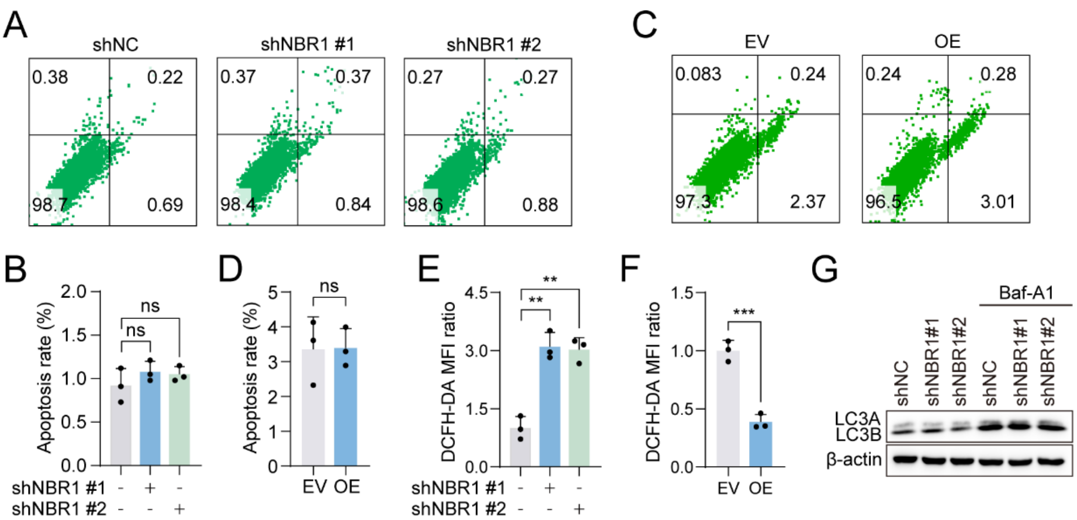

Supplementary Fig. S2 NBR1 regulates senescent phenotypes. (**A** and **B**) Flow cytometry

analysis and quantification for cell apoptosis of NPCs infected with shNC or shNBR1 lentivirus. (**C** and **D**) Flow cytometry analysis and quantification for cell apoptosis of NPCs infected with vector or NBR1 lentivirus. Quantification of DCFH-DA staining for DCFH-DA staining of NBR1 silence (**E**) or overexpression (**F**) NPCs. (**G**) LC3A/B protein level in NBR1 silence NPCs treated with or without Baf-A1.

Supplementary Fig. S3

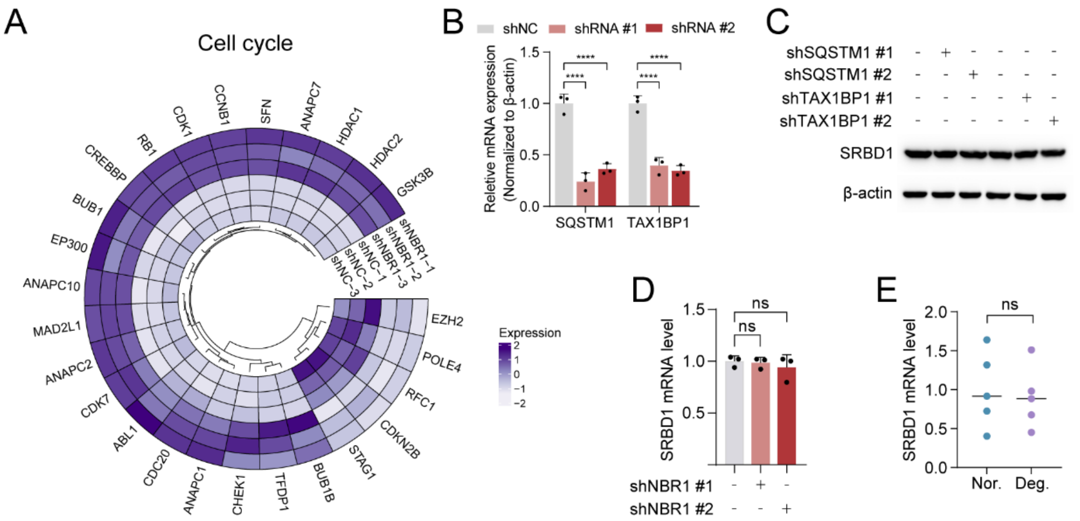

Supplementary Fig. S3 NBR1 retards NPCs senescence by regulating SRBD1. (**A**) Circos plot comparing differentially expressed proteins in NPCs after transfected with shNBR1 lentivirus, highlighting proteins related to cell cycle. (**B**) SQSTM1, TAX1BP1 mRNA levels of NPCs transfected with corresponding shRNAs, as determined by qRT-PCR. (**C**) SQSTM1, TAX1BP1 protein levels of NPCs transfected with corresponding shRNAs, as determined by western blot. (**D**) SRBD1 mRNA expression level in NBR1 silence NPCs. (**E**) SRBD1 mRNA level of NP tissues obtained from patients in normal (n=5) or degenerated group (n=5).

Supplementary Fig. S4

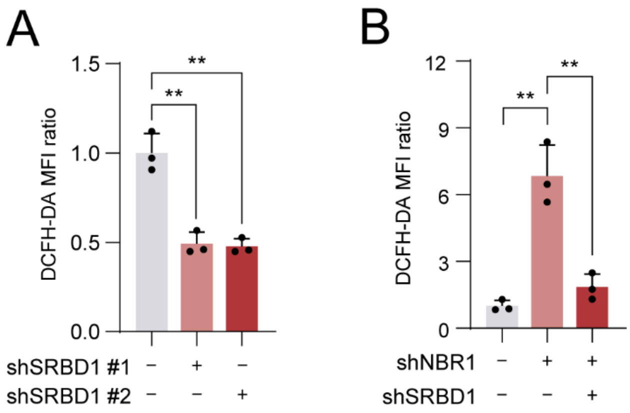

Supplementary Fig. S4 NBR1 regulates the senescent phenotype of NPCs through modulation of SRBD1. (**A**) Quantification of flow cytometry for DCFH-DA staining of NPCs infected with shNC or shSRBD1 lentivirus. (**B**) Quantification of flow cytometry for DCFH-DA staining of NPCs in various experimental groups.

Supplementary Fig. S5

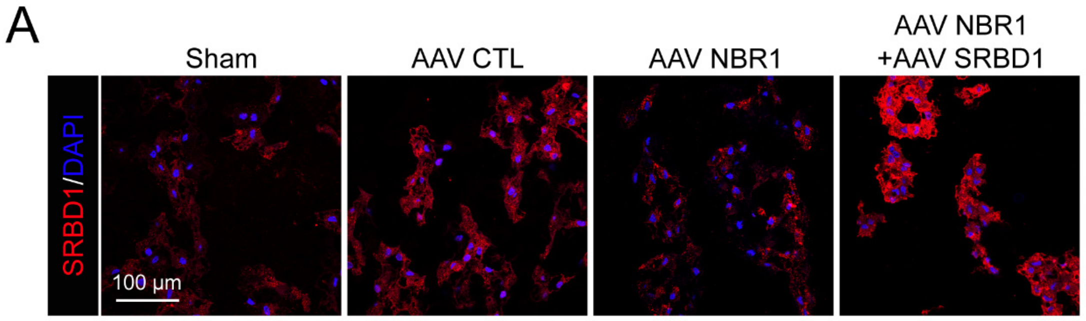

Supplementary Fig. S5 NBR1/SRBD1 axis regulates NPC senescence and IDD progression in *vivo*. (**A**) Representative images of rat coccygeal sections immunostained with SRBD1.

Table S1 Primer sequences for mRNA detection

**Name**	**Forward (5′→3′)**	**Reverse (5′→3′)**
NBR1	TTCCAGAACGCCCGAGCCAA	GGGGCTAGTCAAGTCTGTC
ACAN	GTGCCTATCAGGACAAGGTCT	GATGCCTTTCACCACGACTTC
ADAMTS4	GTCCCATGTGCAACGTCAAG	ATGCGGCCATCTTGTCATCT
ADAMTS5	GGGCACTGGCTACTATGTGG	CGTCACAGCCAGTTCTCACA
MMP13	TCGGCCACTCCTTAGGTCTT	AAGTGGCTTTTGCCGGTGTA
P16	GATCCAGGTGGGTAGAAGGTC	CCCCTGCAAACTTCGTCCT
P21	TGTCCGTCAGAACCCATGC	AAAGTCGAAGTTCCATCGCTC
IL-1β	CCACAGACCTTCCAGGAGAATG	GTGCAGTTCAGTGATCGTACAGG
IL-6	AGACAGCCACTCACCTCTTCAG	TTCTGCCAGTGCCTCTTTGCTG
AKT1	TGGACTACCTGCACTCGGAGAA	GTGCCGCAAAAGGTCTTCATGG
CDK1	GGAAACCAGGAAGCCTAGCATC	GGATGATTCAGTGCCATTTTGCC
EP300	GATGACCCTTCCCAGCCTCAAA	GCCAGATCTCATGGTGAAGG
HDAC1	GGTCCAAATGCAGGCGATTCCT	TCGGAGAACTCTTCCTCACAGG
CCNB1	GACCTGTGTCAGGCTTTCTCTG	GGTATTTTGGTCTGACTGCTTGC
CDC20	CGGAAGACCTGCCGTTACATTC	CAGAGCTTGCACTCCACAGGTA
EZH2	GACCTCTGTCTTACTTGTGGAGC	CGTCAGATGGTGCCAGCAATAG
β-actin	CACCATTGGCAATGAGCGGTTC	AGGTCTTTGCGGATGTCCACGT

Table S2 Antibodies information

Antibodies	Company	Catalog #	Application/Dilution
NBR1	Santa Cruz	sc-130380	WB (1:200); IHC (1:50); IF (1:50)
ACAN	Proteintech	13880-1-AP	WB (1:1000); IF (1:500)
ADAMTS4	Abcam	ab185722	WB (1:1000)
ADAMTS5	Abcam	ab41037	WB (1:1000)
MMP13	Abcam	Ab39012	WB (1:1000)
p21	Proteintech	10355-1-AP	WB (1:1000)
p16	Santa Cruz	sc-51243	WB (1:100); IF (1:50)
SRBD1	Sigma	SAB1407615	WB (1:1000)
p53	Abcam	ab26	WB (1:1000)
AKT1	CST	2938	WB (1:1000)
p-p65	CST	3033	WB (1:1000)
p65	CST	3034	WB (1:1000)
RB	Abcam	ab181616	WB (1:1000)
β-actin	HUABIO	M1210-2	WB (1:5000)

Table S3. Detailed information for 32 proteins identified by 4D label-free proteomic and IP-MS (ranked by fold change)

Number	Accession	Description	Fold change
1	Q8N5C6	SRBD1_HUMAN S1 RNA-binding domain-containing protein 1 OS=Homo sapiens OX=9606 GN=SRBD1 PE=1 SV=2	50.63543978
2	A0A0J9YWL9	TX13C_HUMAN Putative testis-expressed protein 13C OS=Homo sapiens OX=9606 GN=TEX13C PE=5 SV=1	14.02979976
3	O95251	KAT7_HUMAN Histone acetyltransferase KAT7 OS=Homo sapiens OX=9606 GN=KAT7 PE=1 SV=1	5.224938763
4	Q9BRZ2	TRI56_HUMAN E3 ubiquitin-protein ligase TRIM56 OS=Homo sapiens OX=9606 GN=TRIM56 PE=1 SV=3	3.598650766
5	Q16384	SSX1_HUMAN Protein SSX1 OS=Homo sapiens OX=9606 GN=SSX1 PE=1 SV=2	3.399472561
6	O95425	SVIL_HUMAN Supervillin OS=Homo sapiens OX=9606 GN=SVIL PE=1 SV=2	2.793122259
7	Q9NQ29	LUC7L_HUMAN Putative RNA-binding protein Luc7-like 1 OS=Homo sapiens OX=9606 GN=LUC7L PE=1 SV=1	2.737533548
8	Q9UQ88	CD11A_HUMAN Cyclin-dependent kinase 11A OS=Homo sapiens OX=9606 GN=CDK11A PE=1 SV=4	2.684602714
9	Q96T88	UHRF1_HUMAN E3 ubiquitin-protein ligase UHRF1 OS=Homo sapiens OX=9606 GN=UHRF1 PE=1 SV=1	2.568367809
10	Q9HC07	TM165_HUMAN Transmembrane protein 165 OS=Homo sapiens OX=9606 GN=TMEM165 PE=1 SV=1	2.562645777
11	Q96NE9	FRMD6_HUMAN FERM domain-containing protein 6 OS=Homo sapiens OX=9606 GN=FRMD6 PE=1 SV=1	2.557427654
12	P46013	KI67_HUMAN Proliferation marker protein Ki-67 OS=Homo sapiens OX=9606 GN=MKI67 PE=1 SV=2	2.526341421
13	Q01804	OTUD4_HUMAN OTU domain-containing protein 4 OS=Homo sapiens OX=9606 GN=OTUD4 PE=1 SV=4	2.506026463

14	Q9HAN9	NMNA1_HUMAN Nicotinamide/nicotinic acid mononucleotide adenylyltransferase 1 OS=Homo sapiens OX=9606 GN=NMNAT1 PE=1 SV=1	2.462463804
15	P46778	RL21_HUMAN 60S ribosomal protein L21 OS=Homo sapiens OX=9606 GN=RPL21 PE=1 SV=2	2.447680516
16	Q9UH17	ABC3B_HUMAN DNA dC->dU-editing enzyme APOBEC-3B OS=Homo sapiens OX=9606 GN=APOBEC3B PE=1 SV=1	2.39955418
17	O43660	PLRG1_HUMAN Pleiotropic regulator 1 OS=Homo sapiens OX=9606 GN=PLRG1 PE=1 SV=1	2.391817094
18	Q9H9B1	EHMT1_HUMAN Histone-lysine N-methyltransferase EHMT1 OS=Homo sapiens OX=9606 GN=EHMT1 PE=1 SV=4	2.372888048
19	Q15800	MSMO1_HUMAN Methylsterol monooxygenase 1 OS=Homo sapiens OX=9606 GN=MSMO1 PE=1 SV=1	2.249218587
20	Q8N684	CPSF7_HUMAN Cleavage and polyadenylation specificity factor subunit 7 OS=Homo sapiens OX=9606 GN=CPSF7 PE=1 SV=1	2.220114633
21	O75533	SF3B1_HUMAN Splicing factor 3B subunit 1 OS=Homo sapiens OX=9606 GN=SF3B1 PE=1 SV=3	2.186060336
22	P82675	RT05_HUMAN 28S ribosomal protein S5, mitochondrial OS=Homo sapiens OX=9606 GN=MRPS5 PE=1 SV=2	2.161491677
23	O15226	NKRF_HUMAN NF-kappa-B-repressing factor OS=Homo sapiens OX=9606 GN=NKRF PE=1 SV=2	2.13815002
24	Q86YT6	MIB1_HUMAN E3 ubiquitin-protein ligase MIB1 OS=Homo sapiens OX=9606 GN=MIB1 PE=1 SV=1	2.124131404
25	Q15334	L2GL1_HUMAN Lethal(2) giant larvae protein homolog 1 OS=Homo sapiens OX=9606 GN=LLGL1 PE=1 SV=3	2.077573619
26	Q02543	RL18A_HUMAN 60S ribosomal protein L18a OS=Homo sapiens OX=9606 GN=RPL18A PE=1 SV=2	2.072391233
27	P50402	EMD_HUMAN Emerin OS=Homo sapiens OX=9606 GN=EMD PE=1 SV=1	2.060547814
28	P08621	RU17_HUMAN U1 small nuclear ribonucleoprotein 70 kDa OS=Homo sapiens	2.05489978

		OX=9606 GN=SNRNP70 PE=1 SV=2	
29	P43355	MAGA1_HUMAN Melanoma-associated antigen 1 OS=Homo sapiens OX=9606 GN=MAGEA1 PE=1 SV=1	2.046375095
30	Q9BQ39	DDX50_HUMAN ATP-dependent RNA helicase DDX50 OS=Homo sapiens OX=9606 GN=DDX50 PE=1 SV=1	2.042845041
31	Q9Y4P3	TBL2_HUMAN Transducin beta-like protein 2 OS=Homo sapiens OX=9606 GN=TBL2 PE=1 SV=1	2.026773541
32	P10301	RRAS_HUMAN Ras-related protein R-Ras OS=Homo sapiens OX=9606 GN=RRAS PE=1 SV=1	2.002732076

72
